# Supplementary material for: Circulating tRNA-Derived Small RNAs as Novel Radiation Biomarkers of Heavy Ion, Proton and X-ray Exposure
Source: Int J Mol Sci. 2021 Dec 15;22(24):13476. doi: 10.3390/ijms222413476 (PMC8706565; doi:10.3390/ijms222413476)
Supplement: Supplementary file 1 [file ijms-22-13476-s001.zip › ijms-1474994-supplementary.pdf]

Supplementary materials:

Circulating tRNA-derived small RNAs as novel radiation biomarkers of heavy ion, proton and X-ray exposure

Authors

Wenjun Wei[1,2,#], Hao Bai[1,2,#], Yaxiong Chen[1], Tongshan Zhang[1,2], Yanan Zhang[1], Junrui Hua[1], Jinpeng He[1,2], Nan Ding[1,2], Heng Zhou[1,2], Jufang Wang[1,2,*]

1. Key Laboratory of Space Radiobiology of Gansu Province & CAS Key Laboratory of Heavy Ion Radiation Biology and Medicine, Institute of Modern Physics, Chinese Academy of Sciences, Lanzhou 730000, China.
2. University of Chinese Academy of Sciences, Beijing 100049, China

*Correspondence to:

Jufang Wang

Institute of Modern Physics, Chinese Academy of Sciences

509 Nanchang Road, Lanzhou 730000, China

Tel: +86-0931-5196184
E-mail address: jufangwang@impcas.ac.cn

Supplementary Figures

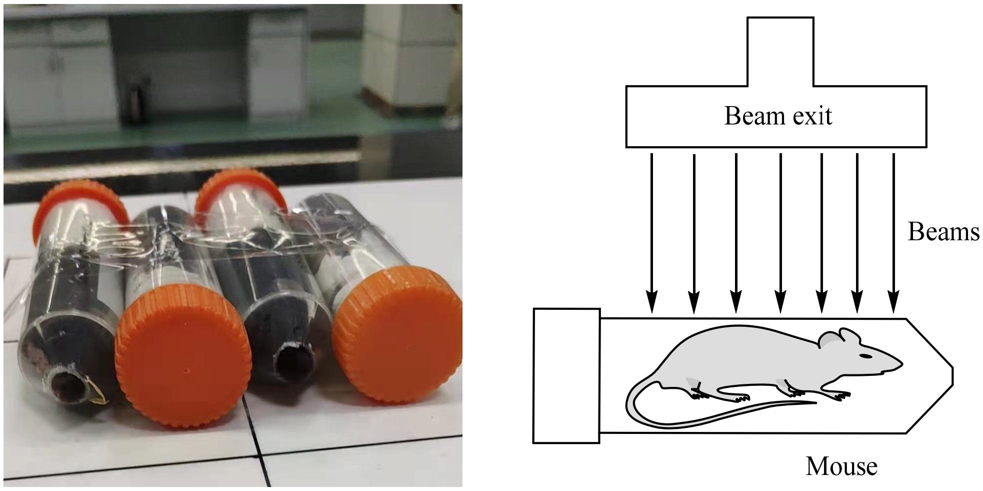

Supplementary Figure S1: The sketch to present the irradiation geometry of mouse.

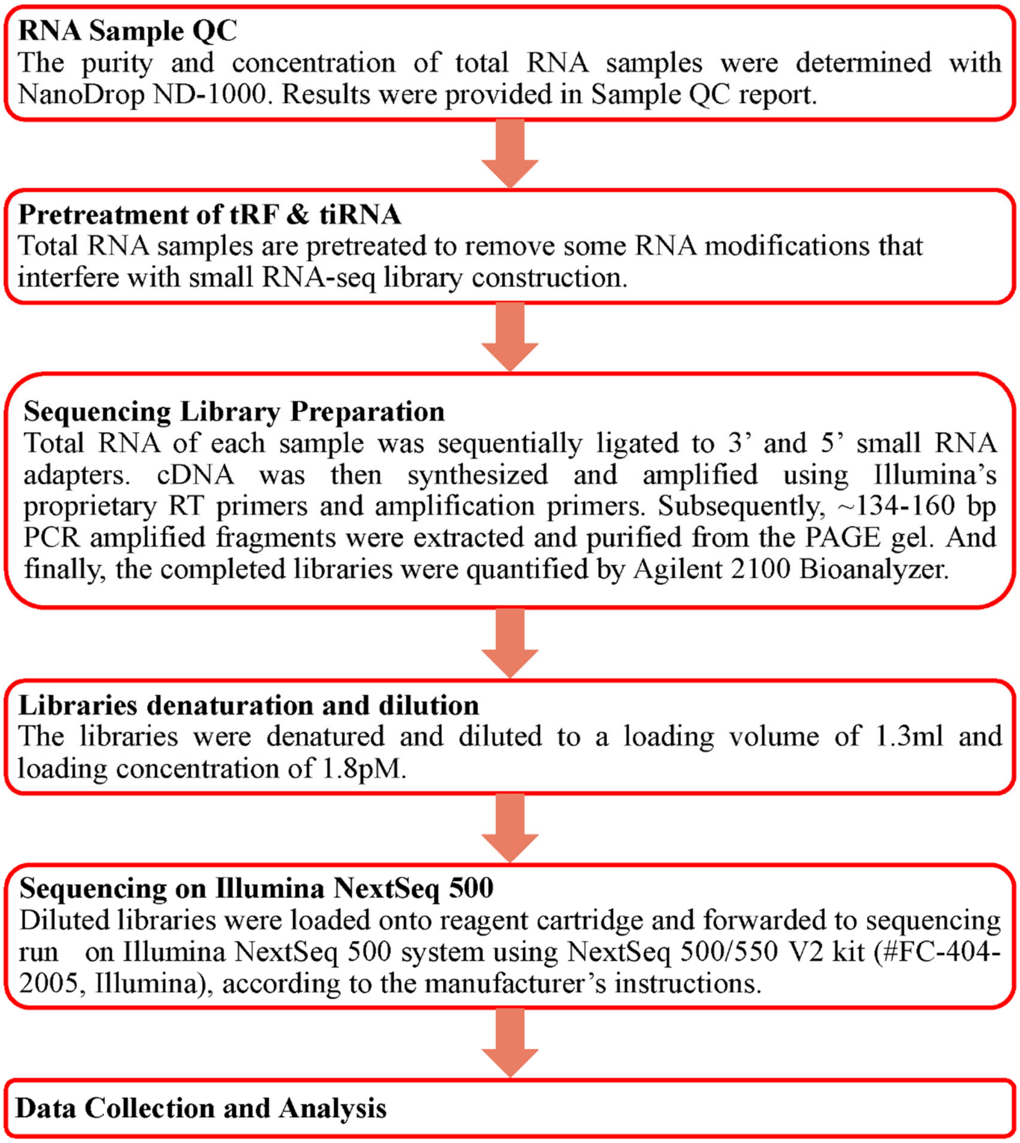

Supplementary Figure S2: The overview flowchart of RNA-sequencing.

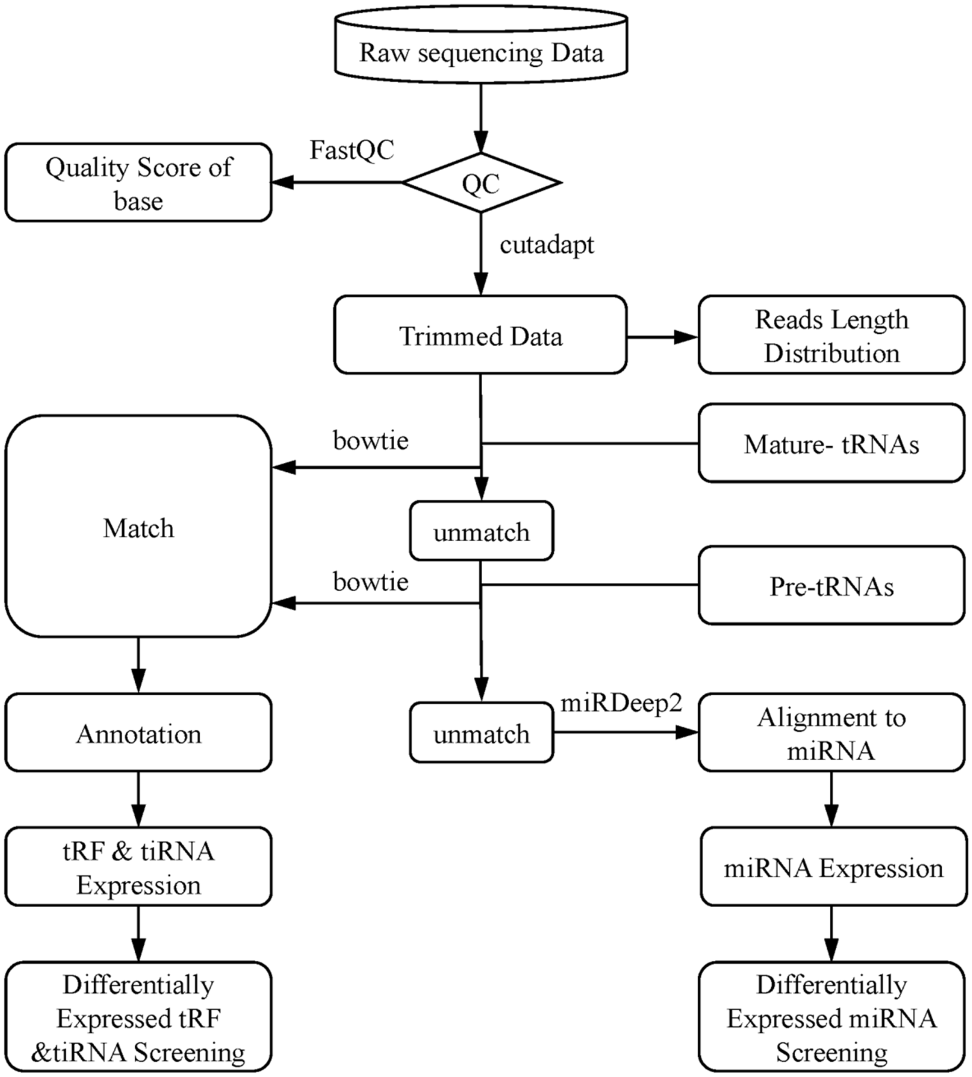

Supplementary Figure S3: The workflow of RNA-seq data analysis.

Supplemental Tables

Supplemental Table S1. Mapping summary of small RNA reads in serum of mice exposed to different doses of carbon ions.

Dose	Trimmed number	Mature tRNA	Mature tRNA (%)	Pre-tRNA	Pre-tRNA (%)	miRNA	miRNA (%)
0 Gy	8898955	6777843	76.16	43069	0.48	283157	3.18
0.05 Gy	8687701	7048102	81.13	32361	0.37	44684	0.51
0.1 Gy	8808837	7328342	83.19	3875	0.04	19328	0.22
0.5 Gy	8924739	7233239	81.05	46074	0.52	59126	0.66
1 Gy	9149154	7488450	81.85	38509	0.42	79925	0.87

Supplemental Table S2: The expression data of 10 selected tsRNA from RNA sequencing.

tsRNA ID	CMP					Relative expression (compared to 0 Gy)			
	0Gy	0.05Gy	0.1Gy	0.5Gy	1Gy	0.05 Gy	0.1 Gy	0.5 Gy	1 Gy
tRF-Glu-TTC-023	3604.63	1770.89	155.02	1750.68	1297.55	0.59	0.44	0.52	0.59
tiRNA-Glu-TTC-003	358.13	160.52	5.06	170.87	45.87	0.54	0.15	0.51	0.21
tRF-Val-AAC-024	2190.69	947.58	64.52	707.40	596.43	0.52	0.30	0.35	0.45
tRF-Glu-TTC-029	1998.15	661.93	54.41	823.32	343.75	0.40	0.28	0.44	0.28
tRF-Gln-TTG-013	108.70	236.03	7.38	274.49	166.34	2.62	0.70	2.70	2.52
tRF-Met-CAT-006	244.04	506.01	102.39	512.62	379.36	2.50	4.32	2.24	2.56
tRF-Gln-CTG-018	1053.57	1900.92	60.70	2764.46	1220.65	2.17	0.59	2.80	1.91
tRF-Lys-CTT-008	59.59	29.34	2.19	30.90	21.59	0.59	0.38	0.55	0.60
tRF-Lys-TTT-019	358.13	160.52	5.06	170.87	45.87	0.54	0.15	0.51	0.21
tRF-Gln-CTG-019	172.18	233.88	316.02	316.02	192.24	1.64	0.56	1.96	1.84

CMP：the counts per million of total aligned reads.

Supplemental Table S3: The mature sequences of the 5 tsRNA biomarkers and their primers designed for RT-qPCR.

tRF&tiRNA ID	Sequences 5′-3′	Designed primers
tiRNA-Glu -TTC-003	5′-TCCCATATGGTCTAGCGGTTAGGATTC-3′	5′-TCCCATATGGTCTAGCGGTTAGGATTC-3′
tRF-Val-AAC -024	5′-GTTTCCGTAGTGTAGTGGTTATCACGTTCG-3′	5′-GTTTCCGTAGTGTAGTGGTTATCACGTTCG-3′
tRF-Gln-CTG -018	5′-GGTTCCATGGTGTAATGGTTAGCACTCT-3′	5′-GGTTCCATGGTGTAATGGTTAGCACTCT-3′
tRF-Lys-CTT-008	5′-GCCCGGCTAGCTCAGTC-3′	5′-CTAGCTCAGTCGGTAGAGCATGAG-3′
tRF-Lys-TTT-019	5′-AGCCCGGATAGCTCAGTCG	5′-AGCCCGGATAGCTCAGTCG-3′

Supplemental Table S4: The coefficients of fitting the multiple liner regression based on the dose-response data of 5 tsRNA biomarkers responding to carbon ions, X-rays or protons. Beta values means the partial regression coefficient, which was used to build the multi-factor equation.

**Irradiation types**	**Model**	**Unstandardized Coefficients**		**Standardized Coefficients**	**t**	**Significance**
		**Beta**	**Std. Error**	**Beta**		
Carbon ion irradiation	Constant	0.646	0.130		4.977	0.000
	tiRNA-Glu-TTC-003	-0.149	0.069	-0.459	-2.163	0.035
	tRF-Val-AAC-024	0.022	0.045	0.089	0.480	0.634
	tRF-Gln-CTG-018	-0.093	0.034	-0.294	-2.723	0.009
	tRF-Lys-CTT-008	0.139	0.086	0.301	1.615	0.113
	tRF-Lys-TTT-019	-0.206	0.090	-0.453	-2.294	0.026
X-ray irradiation	Constant	0.943	0.210		4.489	0.000
	tiRNA-Glu-TTC-003	0.000	0.079	-0.003	-0.004	0.997
	tRF-Val-AAC-024	-0.050	0.090	-0.247	-0.553	0.586
	tRF-Gln-CTG-018	-0.053	0.041	-0.335	-1.285	0.214
	tRF-Lys-CTT-008	-0.013	0.062	-0.364	-0.202	0.842
	tRF-Lys-TTT-019	0.023	0.111	0.442	0.206	0.839
Proton irradiation	Constant	0.521	0.213		2.444	0.028
	tiRNA-Glu-TTC-003	-0.081	0.082	-0.753	-0.995	0.336
	tRF-Val-AAC-024	0.788	0.247	3.896	3.194	0.006
	tRF-Gln-CTG-018	-0.077	0.161	-0.415	-0.479	0.639
	tRF-Lys-CTT-008	-0.646	0.435	-2.854	-1.485	0.160
	tRF-Lys-TTT-019	-0.077	0.461	-0.347	-0.166	0.870
